# Immunohistochemical optimization of a panel of antibodies against amyloid precursor protein for immunodetection of axonal injury in neurologic diseases of sheep

**DOI:** 10.1177/03009858261433523

**Published:** 2026-03-25

**Authors:** Jim Manavis, Glyn Chidlow, Peter Blumbergs, Ian Jerrett, John Finnie

**Affiliations:** 1 University of Adelaide, Adelaide, SA, Australia; 2 Agriculture Victoria Research, AgriBio Centre, Bundoora, VIC, Australia

**Keywords:** amyloid precursor protein, antibodies, axonal injury, brain, comparative immunohistochemistry, myelin immunostaining, sheep

## Abstract

Amyloid precursor protein (APP) is the most sensitive early immunomarker of axonal injury in the brain. Currently, there are several commercially available antibodies to detect axonal spheroids expressing APP. However, immunolabeling of a given molecule can be variable when antibodies are directed to different epitopes. In this study, we optimized a panel of antibodies (22C11, Y188, NAB228, C1/6.1) for labeling APP-immunopositive axonal spheroids in ovine brains and compared the patterns of injury in brains containing spheroids from neurotoxic (clostridial focal symmetrical encephalomalacia [FSE]) and traumatic brain injury. The pattern of axonal spheroid immunolabeling in FSE necrotic foci and trauma-damaged white matter was similar with all 4 antibodies. Only subtle differences in axonal immunolabeling were found probably due to whether the C- or N-terminal fragment of APP was targeted. However, while background labeling with 3 of the antibodies was minimal, there was robust, diffuse labeling of axons due to myelin immunolabeling with the Y188 antibody, which was determined to be specific. Thus, while these antibodies can detect axonal injury with similar efficacy in sheep brains, caution must be taken with Y188 owing to confounding myelin immunolabeling. This study will hopefully serve as a guide for optimal immunodetection of axonal injury in ovine encephalopathies.

A panel of antibodies is required for immunodetection of the full range of heterogeneous axonal injury in the brain after an insult. In ovine brains, a recent study^9^ showed that axonal swellings (spheroids) were best detected by an antibody to amyloid precursor protein (APP). The most sensitive early immunomarker of axonal injury is APP,^11^ which enables detection of axonal damage within 30 minutes of an initial insult, probably even after an interval of only several minutes.^1^

It is important in diagnostic immunohistochemistry to be confident that the selected antibody accurately and fully detects the cellular element of interest.^2^ However, in addition to variable results produced by a given antibody from different commercial sources and differing antibody dilutions and retrieval methods, immunohistochemistry can potentially be variable when antibodies are directed to different epitopes (antigenic determinants) on a given molecule. In the case of APP, there are several commercially available antibodies directed against different epitopes. Accordingly, we wished to extend our previous study using 22C11 in focal symmetrical encephalomalacia (FSE) lesions^9^ to compare the efficacy and optimal dilution of this antibody with several other antibodies for the detection of APP-immunopositive axonal spheroids.

Axonal spheroids are swellings of the axon that develop in response to focal disruption of the axonal membrane, which permits the influx of calcium and leads to the activation of multiple deleterious, calcium-dependent cascades. The resulting proteolytic destruction of the axonal cytoskeleton disassembles microtubules, upon which fast axonal transport is dependent. When axonal transport is impeded, there is an accumulation of neurofilaments and organelles, which is manifest as axonal spheroids or varicosities.^11^

APP, a constitutively expressed, highly conserved, 100 to 140 kDa transmembrane glycoprotein found in neurons, is normally anterogradely carried along an axon by fast axoplasmic transport as a membrane-bound vesicular protein. When this transport is disrupted, APP accumulates rapidly proximal to the site of injury.^4,7,11^

There are 3 domains of APP, a transmembrane domain, a large extracellular N-terminal domain, and a short cytoplasmic C-terminal tail. Clone 22C11 is a mouse monoclonal antibody targeted to amino acids 66 to 81 of the N-terminus on APP^5^ that recognizes all major isoforms of APP. Clone Y188 is a rabbit monoclonal antibody that recognizes the YENPTY motif that is located in the final 15 amino acids of the C-terminal region of APP.^6^ Y188 detects full-length APP as well as its C-terminal fragments. Clone C1/6.1 is a mouse monoclonal antibody that targets the C-terminal fragment of APP, which recognizes full-length APP as well as its C-terminal fragments. Clone NAB228 is a mouse monoclonal antibody directed to the N-terminal fragment, which detects full-length APP, the soluble APP-alpha form, and Aβpeptides. Since the amount of APP in undamaged axons is insufficient to be detected by routine light microscopy, APP only labels injured axons.^2,5^ When APP accumulates due to disruption of fast axoplasmic transport, axonal spheroids can become readily visible in histologic sections less than 35 minutes after the initial insult.^1,11^

The relative ability of these antibodies to detect APP-immunoreactive axonal injury in the brain was evaluated using brain lesions in sheep produced *by Clostridium perfringens* type D epsilon toxin (termed FSE) and traumatic brain injury. FSE is the chronic form of epsilon enterotoxemia, which occurs when lower doses of toxin are absorbed into the systemic circulation from the small intestine or if sheep are partially immune, with the resulting neurologic disease following a more protracted clinical course.^3,12^ Brain samples from 4 sheep (2-, 2-, 3-, and 4-year-old Merino-Border Leicester ewes) with naturally occurring FSE were obtained from Agriculture Victoria Research, Melbourne, Australia, while brains from sheep with traumatic injury were collected from four 2-year-old Merino ewes subjected to an experimental model of impact-acceleration head injury, as previously described.^8^ Briefly, anesthetized and ventilated Merino ewes sustained an impact to the temporal region of an unrestrained head with a humane stunner. Six hours post-impact under anesthesia, the brain was perfusion-fixed with 4% paraformaldehyde. All brains were then immersion-fixed in 10% neutral-buffered formalin, paraffin-embedded as coronal sections, and 6 µm sections were cut and stained with hematoxylin and eosin. Duplicate sections were cut for immunohistochemical analysis. Four 2-year-old Merino-Border Leicester ewes, which were clinically normal and free of any brain lesions when stained with hematoxylin and eosin, were used as control animals. This study was approved by the Animal Ethics Committees of the Royal Adelaide Hospital, University of Adelaide, and Institute of Medical and Veterinary Science.

For colorimetric immunohistochemistry, the following anti-APP antibodies were used: mouse monoclonal, clone 22C11 (gift from Professor Colin Masters; dilutions tested: 1:500, 1:1000, 1:1500, 1:4500); rabbit monoclonal, clone Y188 (Abcam, cat. Ab32136; concentrations tested: 0.768 µg/ml, 0.256 µg/ml, 0.085 µg/ml, 0.0384 µg/ml, 0.0128 µg/ml); mouse monoclonal, clone C1/6.1 (Biolegend, cat. 802801; concentrations tested: 4 µg/ml, 2 µg/ml, 1 µg/ml, 0.225 µg/ml); and a mouse monoclonal NAB228 (Cell Signaling Technology, cat. 2450; concentrations tested: 3.3 µg/ml, 1.1 µg/ml, 0.369 µg/ml). A polyclonal rabbit anti-myelin basic protein (MBP) (Dako, cat. A-0623; 1:5000) was also used to detect myelin. In brief, sections were dewaxed using xylene and then dehydrated through alcohols. Sections were treated with methanol/hydrogen peroxide for 30 minutes. All sections were then rinsed twice in phosphate-buffered saline (PBS; pH 7.4) for a further 5 minutes each wash. Antigen retrieval was performed using citrate buffer (pH 6). Slides were allowed to cool and washed twice in PBS (pH 7.4). Non-specific proteins were blocked using 3% normal horse serum for 30 minutes. Antibodies were then applied at the above-listed dilutions at room temperature overnight. The following day, the sections were given 2 washes in PBS and then incubated consecutively with biotinylated secondary antibody (1:250; Vector Laboratories) for 30 minutes and streptavidin-peroxidase conjugate (1:1000; Thermo Scientific) for 1 hour. Sections were then visualized using diaminobenzidine tetrahydrochloride, washed, counterstained with hematoxylin, dehydrated, cleared, and mounted on glass slides. Specificity of labeling was judged by the morphology and distribution of the labeled cells, by the absence of signal when the primary antibody was replaced by isotype/serum controls (negative controls), and by comparison with the expected labeling pattern based on our own, and other, previously published results. For double-labeling immunofluorescence of Y188 with MBP, visualization of Y188 was achieved using a 3-step procedure (Y188, then biotinylated anti-rabbit secondary antibody, then streptavidin-conjugated AlexaFluor 594, Thermo Scientific), while MBP was labeled by a 2-step procedure (mouse anti-MBP primary antibody, Santa Cruz, cat. 271524, then anti-mouse secondary antibody conjugated to AlexaFluor 488, Thermo Scientific).

Overall, the patterns of axonal spheroid immunopositivity obtained with each of the 4 anti-APP antibodies were similar in necrotic FSE foci, which were found in the basal ganglia (Fig. 1 a–d), thalamus (data not shown), and cerebellar peduncles (data not shown) and in the widely distributed axonal injury in traumatized hemispheric white matter (Fig. 1 e–h). There were sometimes minor differences in spheroid immunolabeling within some necrotic lesions, which was probably a reflection of the different epitopes detected by the respective antibodies on the APP molecule. In control sheep brains, no APP-immunopositive axonal spheroids were found with any of the 4 anti-APP antibodies tested; however, there was a marked difference in immunolabeling of the brain neuroparenchyma with Y188, with concentrations in the range of 0.768 µg/ml to 0.0384 µg/ml (representing a dilution range of 1:1000 to 1:10,000) showing strong, diffuse immunolabeling of axons, mainly the myelin sheaths (Fig. 2a). In contrast, there was negligible nerve fiber labeling with the other antibodies at a wide range of dilutions. Myelin labeling with Y188 only diminished to a level comparable to that of the other 3 antibodies at a concentration of 0.0128 µg/ml (representing a 1:30,000 dilution; Fig 2b). These results are summarized in Table 1, and representative images of APP-immunopositive axonal injury at optimal dilutions for each anti-APP antibody are shown in Fig. 1 for the FSE and traumatic brain injury cases.

**Figure 1.**
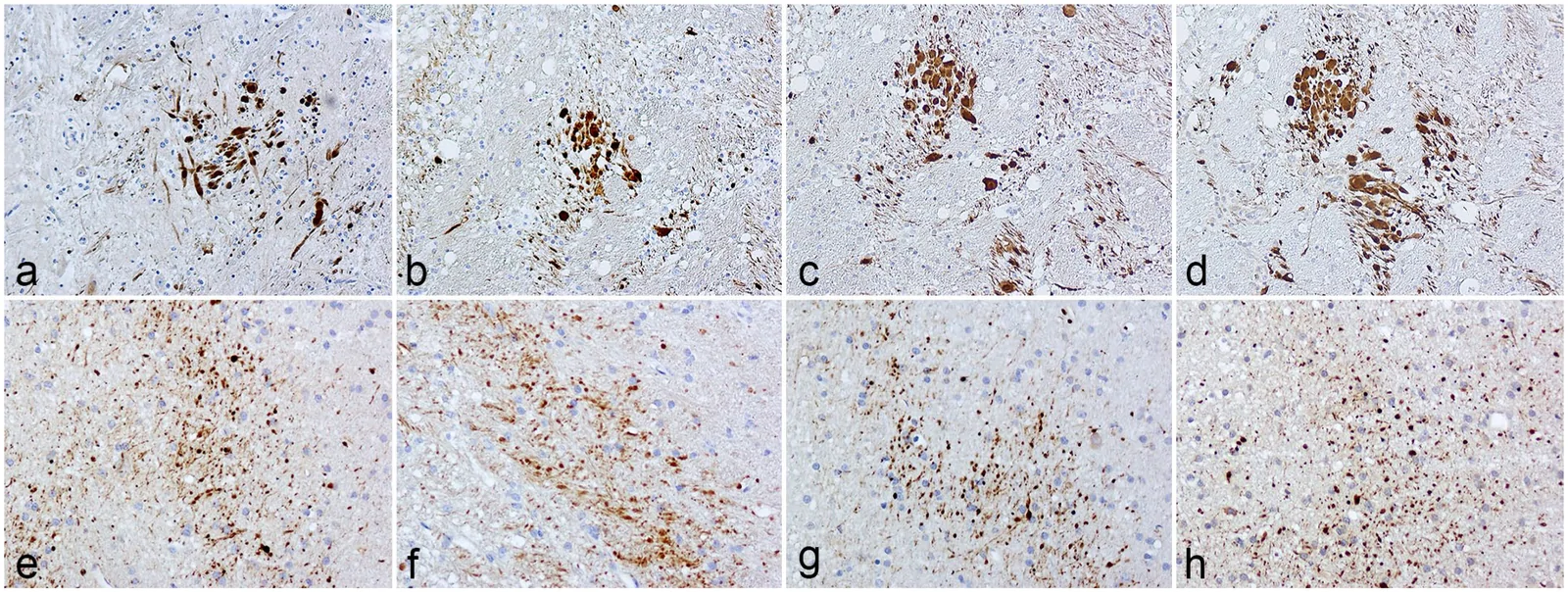
Brain, sheep. Axonal spheroids in a focal symmetrical encephalomalacia lesion in (a–d) the basal ganglia and (e–h) the hemispheric white matter after a traumatic brain injury optimally immunolabeled with (a, e) NAB228 (1:1500 dilution); (b, f) clone C1/6.1 (1:1500 dilution); (c, g) Y188 (1:30000 dilution); and (d, h) clone 22C11 (1:1000 dilution) anti-amyloid precursor protein antibodies.

**Figure 2.**
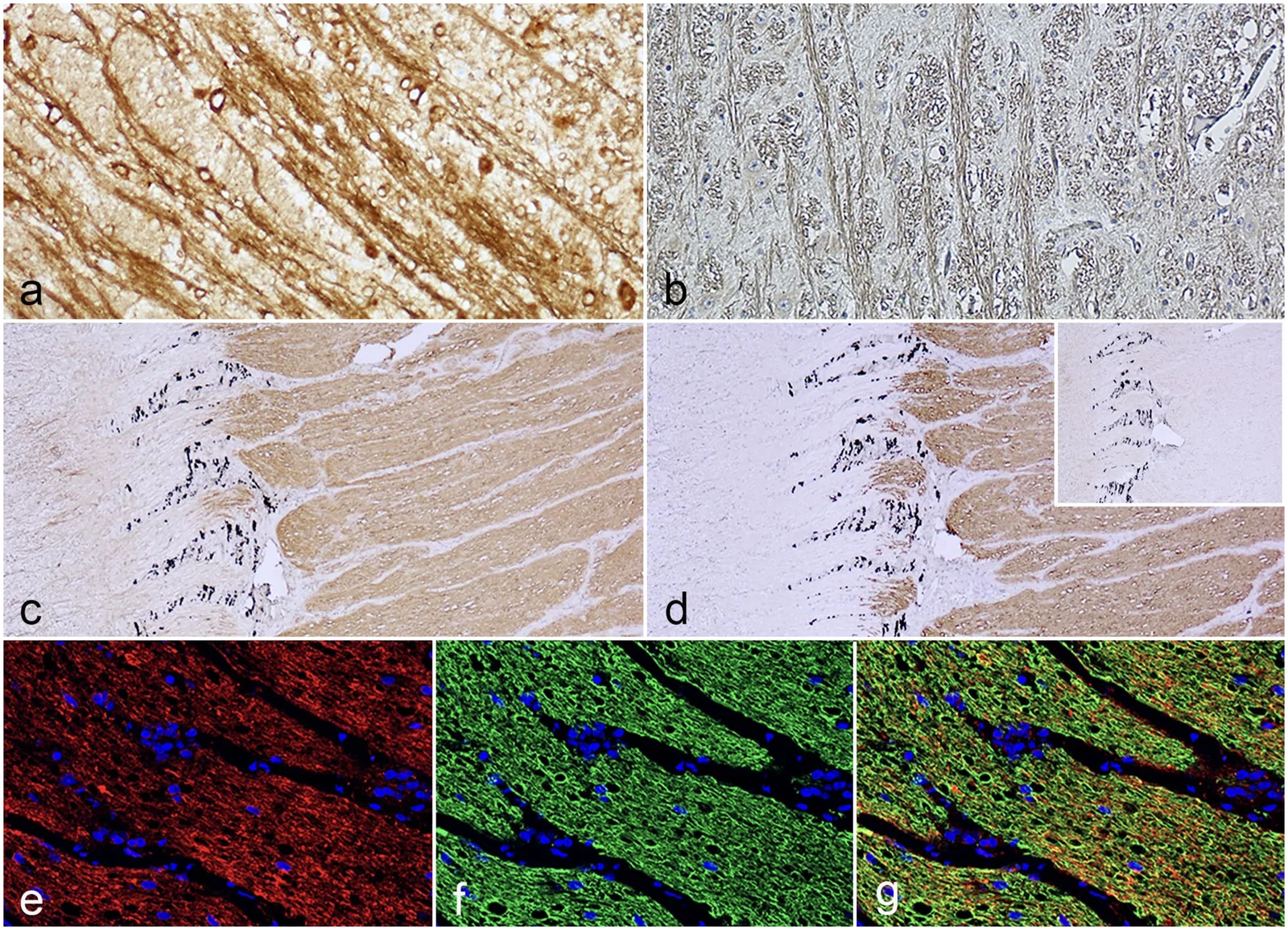
Large myelinated fiber tracts in the sheep brainstem immunolabeled with the Y188 anti-amyloid precursor protein (APP) antibody at (a) 1:2000 and (b) 1:30,000 antibody dilutions. (c) Optic nerve in the sheep immunolabeled with Y188 at 1:2000 antibody dilution. (d) The myelinated portion of the optic nerve was confirmed by a similar immunolabeling pattern with anti-myelin basic protein antibodies (MBP). Inset: No myelin labeling was found with the 22C11 antibody. (e–g) Double-labeling immunofluorescence of APP (Y188) (e) with MBP (f) showed co-localization of APP and MBP (g).

**Table 1.** Summary of anti-APP antibodies used to detect axonal injury in sheep brains.

Anti-APP antibody	Host source	Clonality	APP fragment targeted	Optimal dilution
22C11	mouse, IgG1	monoclonal	a.a. 66-81 (N-terminus) of APP	1:1000
C1/6.1	mouse, IgG1	monoclonal	a.a. 676-695 (C-terminus) of APP695	1:1000 (1 µg/ml)
NAB228	mouse, IgG2a	monoclonal	a.a. 1-15 (N-terminus) of beta-amyloid	1:4000 (0.41 µg/ml)
Y188	rabbit	monoclonal	C-terminus fragment of APP	1:30 000 (0.0128 µg/ml)

Since there was no immunolabeling of myelin by Y188 in the negative control, the myelin labeling produced by this antibody was determined to be specific labeling^3^ representing antigen-antibody reaction. To further assess whether Y188 labeled myelin, ovine optic nerves from control sheep were used. Retinal ganglion cell axons forming the optic nerves are unmyelinated until they exit the eye through fenestrations in the sclera (termed the lamina cribrosa), since myelinated fibers within the retina would impede the path of incoming light. At concentrations stronger than 0.0128 µg/ml, Y188 only labeled the myelinated portion of the optic nerve (Fig. 2c), permitting a clear distinction between myelinated and unmyelinated nerve fibers. Immunolabeling of myelin by Y188 was confirmed by comparable labeling for MBP (Fig. 2d), a major structural protein of central nervous system myelin. By contrast, there was no myelin immunolabeling with the other 3 antibodies (Fig. 2e). Myelin immunolabeling by Y188 was further demonstrated using double-labeling immunofluorescence, which showed co-localization of Y188 and MBP (Fig. 2 f–h). As in the brain, this myelin labeling was apparent at all concentrations of the Y188 antibody tested with the exception of the most dilute, 0.0128 µg/ml.

APP is found in the axolemma, myelin sheath, and at the nodes of Ranvier of nerves and plays a role in the formation and modulation of myelin.^10,13^ However, while APP is found in myelin, it is present in insufficient quantities to produce the strength and extent of myelin immunolabeling seen with Y188 in sheep brains. Y188 is, therefore, probably binding mainly to another myelin component or components.

In conclusion, despite the differences in recognized epitopes of APP, all 4 antibody immunolabeling patterns were similar in 2 different types of brain injury. However, the concentrations of the respective antibodies required to optimally immunolabel axonal spheroids, while producing minimal labeling of unaffected axons, differed widely. While the 22C1, NAB228, and C1/6.1 antibodies against APP produced a robust signal with minimal background labeling of the neuroparenchyma at a wide range of concentrations, Y188 elicited diffuse immunolabeling of uninjured axons over a large concentration range, mainly due to labeling of myelin sheaths. This strong labeling could make it more difficult to visualize small axonal spheroids and conduct quantitation of axonal injury using color deconvolution techniques. Myelin labeling by Y188 only diminished to a level comparable to the other 3 antibodies at a very high dilution. Thus, all 4 antibodies tested are useful for detection of APP in ovine brain lesions and, with the exception of Y188, provide good specificity for axonal injury at lower antibody dilutions. This study provides the first comparison of a panel of anti-APP antibodies for detection of ovine axonal injury.
